# Diagnostic Challenge of Recurrent Dermatofibrosarcoma Protuberans Mimicking Other Spindle Cell Lesions: Importance of Immunohistochemistry in Diagnosis

**DOI:** 10.7759/cureus.110213

**Published:** 2026-06-03

**Authors:** Manasi Anbazhagan, Kevin Manuel, Renu G'Boy Varghese, Vignesh Kumar, Joseph Manuel

**Affiliations:** 1 Pathology, Pondicherry Institute of Medical Sciences, Puducherry, IND; 2 General Surgery, Pondicherry Institute of Medical Sciences, Puducherry, IND; 3 Radiology, Pondicherry Institute of Medical Sciences, Puducherry, IND

**Keywords:** cd34 immunohistochemistry, dermatofibrosarcoma protruberans, differential diagnosis, locally aggressive tumor, spindle-cell tumor

## Abstract

Dermatofibrosarcoma protuberans (DFSP) is an uncommon, low-grade cutaneous sarcoma with fibroblastic differentiation. It typically shows slow progression but is locally aggressive and has a tendency for recurrence if not adequately excised. Although metastasis is rare, DFSP often infiltrates deeper tissues and may closely resemble other benign spindle cell lesions both clinically and on routine histology, creating diagnostic difficulty. Immunohistochemistry plays a crucial supportive role in assisting with the diagnostic workup.

We report a 54-year-old male who presented with a painless swelling over the right scapular region. The lesion was initially diagnosed in 2019 as a benign spindle cell lesion on core needle biopsy and excision biopsy. He developed a recurrence at the same site in 2022, which was again reported as a spindle cell lesion with differentials including fibromatosis, DFSP. In 2024, the patient returned with a progressively enlarging mass at the same location, following which a wide local excision was performed. Histopathological examination showed features consistent with DFSP and immunohistochemistry revealed diffuse CD34 positivity. The tumor was found to infiltrate skeletal muscle and extend up to the deep surgical margin.

This case highlights the diagnostic pitfalls of DFSP, particularly when it presents as a recurrent spindle cell lesion and mimics benign entities such as fibromatosis. Careful histopathological evaluation supported by immunohistochemistry is essential for timely diagnosis and appropriate surgical management.

## Introduction

Dermatofibrosarcoma protuberans (DFSP) is an uncommon cutaneous soft tissue sarcoma that originates within the dermis and characteristically infiltrates the subcutaneous tissue. Although DFSP is the most common dermal sarcoma, it remains an exceedingly rare malignancy, accounting for approximately 1% of all soft-tissue sarcomas. Population-based registry data demonstrate that the global annual incidence ranges from 3.0 to 9.3 cases per million individuals. The tumor typically exhibits an indolent growth pattern but remains locally aggressive and is highly prone for local recurrence if the surgical margins are inadequate. DFSP predominantly affects young and middle-aged adults and mostly arises from the trunk, followed by the proximal extremities and the head and neck region [1,2].

Clinically, DFSP typically presents as a small, slow-growing plaque or nodule that gradually increases in size over several years. Due to its slow-growing and non-specific clinical presentation, the lesion can be confused with benign tumors such as dermatofibroma, fibromatosis or hypertrophic scar tissue. Histologically, the neoplasm is composed of uniform, monomorphic spindle cells arranged in a characteristic storiform architecture. These tumor cells infiltrate the surrounding subcutaneous adipose tissue, yielding a distinctive honeycomb or lace-like pattern [3].

The primary therapeutic modalities for DFSP consist of wide local excision with sufficient surgical margins or Mohs micrographic surgery. Achieving clear surgical margins is paramount, given that incomplete resection correlates strongly with elevated recurrence rates. Although the overall prognosis remains highly favorable following adequate surgical excision, long-term clinical follow-up is mandatory due to the persistent risk of late recurrence [4,5]. In this report, we present the case of a male patient who experienced multiple local recurrences of a scapular DFSP that was initially diagnosed as a benign spindle cell lesion, underscoring the necessity of meticulous clinicopathological correlation integrated with supportive immunohistochemical evaluation.

## Case presentation

We report a case of recurrent DFSP in a male patient in his 50s who presented with a recurrent, progressively enlarging mass over the right scapular region. The primary lesion had initially been evaluated and excised in 2019 as a benign spindle cell lesion. Clinical examination revealed a large, firm, non-tender mass measuring approximately 26 × 25 cm. There were no overlying skin changes. Core needle and excision biopsies showed features suggestive of a benign spindle cell lesion. Retrospective review of these original slides revealed a bland, hypocellular proliferation of monomorphic spindle cells arranged in loose fascicles without significant atypia, necrosis, or mitotic figures. Immunohistochemistry had not been performed during the primary evaluation, and the margin status of the 2019 excision could not be accurately determined. The patient remained asymptomatic until 2022, when he noticed a recurrent swelling at the same site measuring approximately 6 × 6 cm. Wide local excision was performed, and histopathological examination revealed a recurrent spindle cell lesion with differentials of fibromatosis and DFSP. In 2024, he presented once again with a progressively enlarging swelling over the right scapular region for five months. Clinical examination revealed a well-defined mass measuring 19 × 17 cm arising at the site of the previous surgical scar, with no direct tumor infiltration or ulceration of the overlying skin. Table 1 shows the timeline of this case. 

**Table 1:** Chronological Clinical Trajectory and Diagnostic Evolution of the Case DFSP: dermatofibrosarcoma protuberans; STIR: short-tau inversion recovery

Parameter	2019 Presentation	2022 Recurrence	2024 Recurrence
Clinical Presentation	Initial presentation with a painless, gradually progressive right scapular mass of 7 months duration	Developed a recurrent swelling at the previous surgical scar site of 25 days duration	Presented with a progressively enlarging mass at the surgical site for 5 months.
Size	26 × 25 cm	6 × 6 cm	19 × 17 cm
Imaging findings	Not available	Ultrasound: Well defined smooth marginated heteroechoic lesion of size ~8.2x2.7x2.5cm with few cystic and hyperechoic areas noted in the subcutaneous plane paraspinal aspect (~2 cm) of thoracic spine on the right side	MRI: large well-defined, T1 isointense, T2/STIR heterogeneously hyperintense, heterogeneously enhancing lesion in the subcutaneous plane of upper back region with mass effect
Procedure Type	Core needle biopsy followed by excision biopsy	Wide local excision	Wide local excision with intraoperative release from the thoracic spinous process
Histopathology diagnosis	Benign spindle cell lesion	Recurrent spindle cell lesion. Differentials – Fibromatosis, DFSP	DFSP
Immunohistochemistry	Not performed	Not performed	Positive: CD34 (diffuse), vimentin and low Ki-67 index (3%). Negative: S100, smooth muscle actin, desmin, pan-cytokeratin & β-catenin.
Surgical Margin Status	Not specified	Not specified	Involved/positive deep surgical margin. Peripheral margins free of tumor.
Management & Follow-up	Surgical excision. Patient remained asymptomatic for 3 years.	Wide local excision. Regular clinical monitoring until recurrence.	Patient was advised adjuvant radiotherapy

Ultrasonography revealed a heterogeneously hyperechoic lesion in the area of the right scapula that was indicative of a neoplastic etiology. Magnetic resonance imaging (Figure 1) showed a massive, well-defined lesion in the subcutaneous plane of the upper back, which was isointense on T1-weighted images and hyperintense on T2/short-tau inversion recovery (STIR) sequences and with heterogeneous enhancement and mass effect on the surrounding structures.

**Figure 1:**
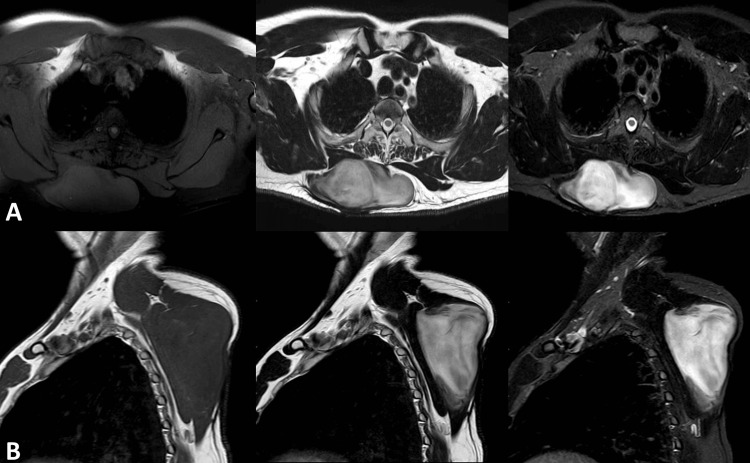
Magnetic resonance imaging of the right scapular region (A) Large well-defined, lobulated, T1 isointense, T2/STIR heterogeneously hyperintense lesion arising from the intramuscular plane of right trapezius muscle extending up to the subcutaneous plane of right upper back region with few central T1/T2/STIR linear hypointense areas. (B) Peripheral T1/T2 hypointense areas are noted in the posterior aspect of the lesion - likely fibrosis STIR: short-tau inversion recovery

Wide local excision was subsequently performed. The resection included the overlying ellipse of skin containing the previous surgical scar. Intraoperatively, the tumor was found to be attached to the spinous process of the thoracic spine. Gross examination (Figure 2) revealed a well-circumscribed, solid, fleshy, pink mass measuring 15.5 × 10.5 × 4 cm. On serial sectioning, the tumor was grossly free from all peripheral surgical margins. The gross clearance of the mass from the respective surgical edges was measured at 8 cm from the lateral margin, 9 cm from the medial margin, 4.5 cm from the superior margin and 5.5 cm from the inferior margin. The deep resected surgical margin appeared involved.

**Figure 2:**
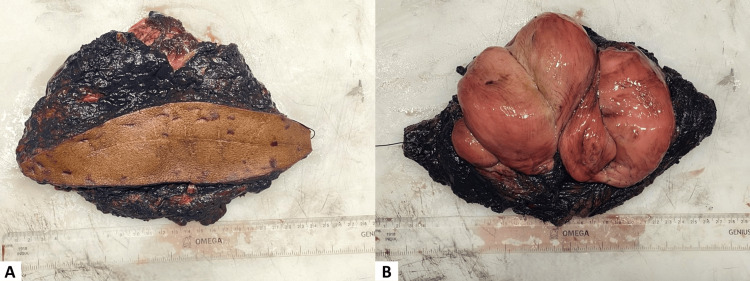
Gross surgical specimen (A) Wide local excision specimen with overlying ellipse of skin. A surgical scar mark is noted on the skin surface. (B) Cut surface shows a well-circumscribed, solid, fleshy pink homogeneous mass measuring 15.5 x 10.5 x 4 cm. At the periphery, the margins of the lesion appear blending with the skeletal muscle fibers. There are no areas of necrosis or hemorrhage.

Histopathological examination (Figure 3) revealed a highly cellular spindle cell neoplasm. Microscopic evaluation of the overlying skin ellipse demonstrated that the epidermis and superficial (papillary) dermis were completely spared and free of tumor. However, the neoplastic cells extended superficially to involve the deep (reticular) dermis immediately underlying the previous surgical scar.

**Figure 3:**
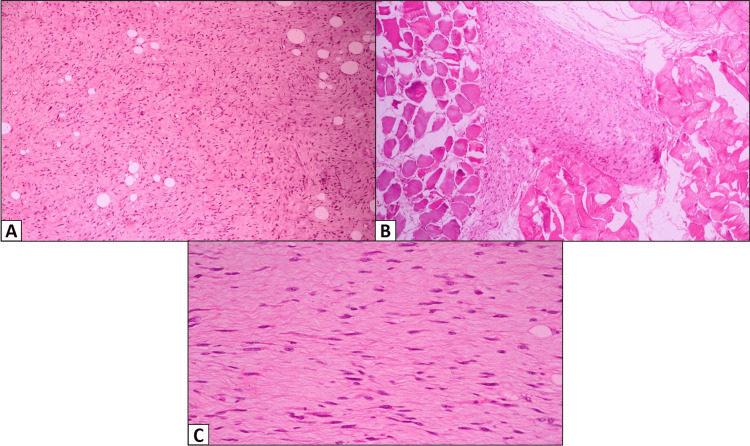
Hematoxylin-eosin staining (A) Spindle cell neoplasm arranged predominantly in fascicles and infiltrating the surrounding adipose tissue (x100) (B) Tumor has infiltrated the adjacent skeletal muscle (x100) (C) Tumor cells have an ovoid to spindled nucleus with indistinct eosinophilic cytoplasm (x400)

The tumor was arranged predominantly in intersecting fascicles, with the classic storiform pattern observed only focally. The tumor demonstrated direct, irregular infiltration into the surrounding adipose tissue (Figure 3A), notably without forming a well-defined, classic honeycomb infiltration of fat. In the deeper sections, the neoplastic infiltration extended directly into the adjacent skeletal muscle (Figure 3B).

At higher magnification, the tumor cells were monomorphic, bland spindle cells featuring ovoid-to-spindle nuclei and indistinct eosinophilic cytoplasm (Figure 3C). Mitotic activity was one per 10 high-power fields (HPF). Crucially, there was absence of significant cellular atypia, pleomorphism, tumor necrosis, atypical mitotic figures and areas of prominent hemorrhage. Furthermore, no herringbone pattern was observed, indicating lack of fibrosarcomatous transformation.

Immunohistochemical analysis (Figure 4) showed strong and diffuse membranous and cytoplasmic immunoreactivity for CD34 involving greater than 95% of the neoplastic spindle cells (Figure 4A). The tumor cells were negative for β-catenin (Figure 4B). Vimentin showed positivity in the tumor cells (Figure 4C). Ki-67 proliferation index was low (3%). The tumor cells were negative for S100, desmin, smooth muscle actin and pan-cytokeratin.

**Figure 4:**
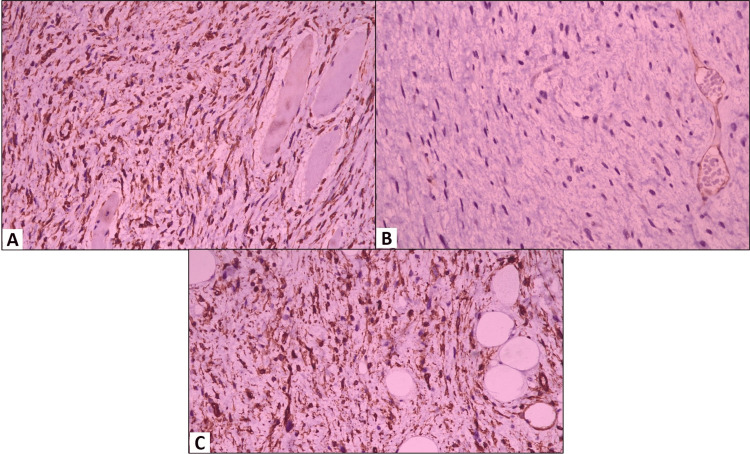
Immunohistochemistry (A) CD34: Tumor cells show strong and diffuse membranous and cytoplasmic positivity (x400) (B) Beta-catenin: Tumor cells are negative (x400) (C) Vimentin: Tumor cells show diffuse cytoplasmic positivity (x400)

The tumor was found to involve the deep surgical margin, while all peripheral margins were free of tumor. Despite the large tumor size, deep skeletal muscle invasion and focal fascicular growth, there was no evidence of fibrosarcomatous transformation.

Based on these findings, a diagnosis of recurrent DFSP without fibrosarcomatous transformation was established. Given the history of multiple recurrences and deep margin involvement, the patient was referred for adjuvant radiotherapy along with close long-term follow-up. However, the patient was subsequently lost to follow-up post-referral.

## Discussion

DFSP is an uncommon cutaneous soft tissue sarcoma that grows slowly, yet with local aggression and a high risk of recurrence when surgical margins are inadequate. Even though the tumor seldom metastasizes, the infiltrative growth tendency results in repeated local recurrence. DFSP usually affects the trunk followed by proximal extremities and the head and neck region, which is consistent with the present case involving the scapular region of the trunk [2,6]. DFSP has a variable clinical presentation and has the potential to cause diagnostic delays. Several studies have reported presentations like atrophic, pigmented and congenital manifestations of DFSP, which can resemble benign dermatologic lesions during the initial phases of the disease. These variants underscore the diverse clinical spectrum of DFSP and emphasize the importance of maintaining a high index of suspicion when evaluating persistent or recurrent cutaneous masses [3,7].

Misdiagnosis of DFSP as other benign spindle cell lesions has been frequently reported in the literature. A case report describing DFSP diagnosed in a primary care setting emphasized that early lesions can be easily mistaken for benign fibrous tumors because of their slow growth and nonspecific clinical appearance [1]. Similarly, studies on recurrent DFSP have highlighted that inadequate sampling or limited biopsy material may lead to misinterpretation as fibromatosis or dermatofibroma [8]. In the present case, the lesion was initially reported as a benign spindle cell lesion. However, the clinical trajectory strongly indicates that the initial 2019 and recurrent 2022 diagnoses did not represent true benign spindle cell lesions. Instead, they represented a single, continuous case of conventional DFSP that was initially under-sampled and misdiagnosed. The definitive diagnosis was established in 2024 only following recurrence and comprehensive histopathological evaluation supported by diffuse CD34 immunoreactivity.

Histopathologically, fibromatosis was considered as one of the differentials based on sweeping fascicles of bland spindle cells and lacking a classic 'honeycomb' pattern of subcutaneous fat infiltration. Our case demonstrated an absolute absence of nuclear β-catenin immunoreactivity, which conclusively excluded fibromatosis.

Histologically, DFSP typically consists of uniform spindle cells arranged in a storiform pattern with infiltration of the subcutaneous fat in a honeycomb pattern. However, these morphological features may overlap with several other spindle cell tumors, making a carefully selected immunohistochemical panel a vital supportive aid in the diagnostic workflow. In the present case, tumor cells showed strong membranous and cytoplasmic expression of CD34 and a low Ki-67 proliferation index (3%), while S100, desmin, SMA, β-catenin and pan-cytokeratin were negative. Though CD34 expression is supportive rather than definitive, evaluating it alongside characteristic morphologic features effectively differentiates DFSP from other spindle cell mimics.

The differential diagnosis of DFSP includes fibromatosis, dermatofibroma, peripheral nerve sheath tumors and smooth muscle tumors. Immunohistochemistry is particularly helpful in distinguishing these entities. Fibromatosis typically demonstrates nuclear β-catenin positivity and absence of CD34 expression, whereas dermatofibroma shows factor XIIIa positivity with weak or negative CD34 staining. Neurogenic tumors such as neurofibroma show strong S100 positivity, while smooth muscle tumors exhibit positivity for SMA and desmin. Diffuse CD34 expression is shared by other mesenchymal lineages such as solitary fibrous tumor (SFT) and superficial CD34-positive fibroblastic tumor (SCPFT). SFT was ruled out by the lack of a "patternless" architecture and staghorn vasculature, while SCPFT was excluded due to the absence of pleomorphic hyperchromatic nuclei and complete negativity for pan-cytokeratin. These morphological and immunohistochemical differences are critical for accurate diagnosis and appropriate management. Table 2 shows the key morphological features and immunohistochemical markers distinguishing various spindle cell lesions.

**Table 2:** Histopathological features and immunohistochemical (IHC) markers distinguishing spindle cell neoplasms DFSP: dermatofibrosarcoma protuberans; SMA: smooth muscle actin; SOX10: SRY-box transcription factor 10; DFSP-FS: dermatofibrosarcoma protuberans with fibrosarcomatous transformation; MPNST: malignant peripheral nerve sheath tumor; EMA: epithelial membrane antigen

Lesion	Key Morphologic Features	Positive IHC Markers	Negative IHC Markers
DFSP	Monotonous storiform proliferation of bland spindle cells with honeycomb subcutaneous fat infiltration	CD34	Cytokeratin, SMA, Desmin, S100, SOX10, β-catenin
Fibromatosis	Bland spindle cells in long sweeping fascicles within abundant collagen with infiltrative margins and low to absent mitotic activity	Nuclear β-catenin, variable SMA	Cytokeratin, Desmin, S100, CD34, SOX10
Dermatofibroma	Proliferation of spindled cells with collagen trapping and epidermal hyperplasia	Factor XIIIa, CD163	Cytokeratin, Desmin, S100, SOX10, CD34
Fibrosarcoma / DFSP-FS	Increased cellularity with cells arranged in long fascicles showing herringbone architecture, nuclear atypia and increased mitoses	Vimentin, variable SMA	Cytokeratin, Desmin, S100, SOX10, β-catenin. CD34 expression is reduced in DFSP-FS areas
MPNST / Neurofibroma	Neurofibroma: Wavy spindle cells with myxoid stroma. MPNST: Hypercellularity and nuclear atypia	S100, SOX10 (diffuse in neurofibroma, patchy and focal in MPNST)	Cytokeratin, SMA, Desmin, β-catenin, CD34
Leiomyosarcoma	Hypercellularity with intersecting fascicles of spindle cells with blunt nuclei, eosinophilic cytoplasm, increased mitotic activity and necrosis	SMA, Desmin, h-caldesmon	Cytokeratin, S100, CD34, SOX10, β-catenin
Spindle cell carcinoma	Fascicles of pleomorphic spindle cells with high mitotic activity and necrosis	Pan-cytokeratin, EMA	Desmin, S100, SOX10, β-catenin, CD34

The recent developments in molecular pathology have also enhanced the insight into the pathogenesis of DFSP. A characteristic COL1A1-PDGFB fusion gene, which is caused by chromosomal translocation and constitutive activation of platelet-derived growth factor signaling pathways, is presented in most cases [9,10]. Diagnostic and therapeutic significance of this genetic alteration has been found in a number of recent molecular studies of DFSP. 

Several studies have reported that certain variants of DFSP may exhibit more aggressive behavior. Among these, fibrosarcomatous transformation is associated with a higher growth rate, increased risk of local recurrence and distant metastasis [11,12]. In addition, long-standing or neglected tumors may grow to a considerable size and subsequently pose significant surgical challenges [13,14]. Despite the tumor's large size and recurrent nature in this case, fibrosarcomatous transformation was ruled out based on the classic histomorphology and diffuse CD34 immunoreactivity.

The primary treatment for DFSP remains wide local excision with adequate surgical margins, but it is associated with higher recurrence rates. In contrast, specialized modalities such as Mohs micrographic surgery are crucial in recurrent DFSP by precisely removing the cancerous tissue while preserving the healthy tissue. Real-time intraoperative margin evaluation serves to minimize the incidence of microscopically positive margins and subsequent local disease persistence [5]. The histopathological study in the current case also showed that the tumor had invaded the nearby skeletal muscle and the deep surgical margin. Based on the history of recurrence rates and the positive margin, adjuvant radiotherapy and close clinical observation were advised.

Systemic targeted therapy with the tyrosine kinase inhibitor imatinib mesylate serves as a therapeutic strategy for patients with unresectable, recurrent, or metastatic disease. Imatinib directly disrupts the autocrine signaling pathway driven by the characteristic COL1A1-PDGFB gene fusion loop, thereby achieving disease control and significant tumor reduction in patients [15].

A limitation of this case report is the lack of molecular testing for the COL1A1-PDGFB gene fusion, which was unavailable at our facility. While molecular confirmation is the gold standard, the diagnosis of DFSP was established based on the combination of focal storiform zones, subcutaneous tissue and musculoskeletal invasion, and strong, diffuse (>95%) membranous and cytoplasmic CD34 immunoreactivity.

## Conclusions

DFSP is a locally aggressive intermediate-grade tumor whose morphological overlap with other spindle cell lesions frequently causes diagnostic delays. The present case underscores these diagnostic complexities, highlighting that recurrent or previously incompletely characterized spindle-cell lesions require a systematic approach. It is essential that these challenging tumors undergo adequate histopathological sampling, precise immunohistochemical profiling, and meticulous margin assessment. This will help ensure diagnostic precision and mitigate the high risk of treatment failure. Ultimately, given the well-documented propensity of infiltrative spindle-cell tumors for delayed recurrence, long-term surveillance remains essential for sustained disease control.
